# Selective DUV Femtosecond Laser Annealing for Electrical Property Modulation in NMOS Inverter

**DOI:** 10.3390/nano15161247

**Published:** 2025-08-14

**Authors:** Joo Hyun Jeong, Won Woo Lee, Sang Jik Kwon, Min-Kyu Park, Eou-Sik Cho

**Affiliations:** 1 Department of Semiconductor Engineering, Gachon University, Seongnam City 13120, Republic of Korea; jjh042512@gachon.ac.kr (J.H.J.); gmr88@gachon.ac.kr (W.W.L.); 2 Department of Electronic Engineering, Gachon University, Seongnam City 13120, Republic of Korea; sjkwon@gachon.ac.kr

**Keywords:** thin film transistor (TFT), zinc oxynitride (ZnON), deep ultraviolet femtosecond (DUV fs) laser annealing, NMOS inverter

## Abstract

Amorphous indium gallium zinc oxide (a-IGZO) is widely used as an oxide semiconductor in the electronics industry due to its low leakage current and high field-effect mobility. However, a-IGZO suffers from notable limitations, including crystallization at temperatures above 600 °C and the high cost of indium. To address these issues, nitrogen-doped zinc oxynitride (ZnON), which can be processed at room temperature, has been proposed. Nitrogen in ZnON effectively reduces oxygen vacancies (V_O_), resulting in enhanced field-effect mobility and improved stability under positive bias stress (PBS) compared to IGZO. In this study, selective deep ultraviolet femtosecond (DUV fs) laser annealing was applied to the channel region of ZnON thin-film transistors (TFTs), enabling rapid threshold voltage (Vth) modulation within microseconds, without the need for vacuum processing. Based on the electrical characteristics of both Vth-modulated and pristine ZnON TFTs, an NMOS inverter was fabricated, demonstrating reliable performance. These results suggest that laser annealing is a promising technique, applicable to various logic circuits and electronic devices.

## 1. Introduction

Metal oxide semiconductors have been widely explored for their excellent electrical properties and transparency, making them attractive for applications in displays, sensors, and logic devices. Among them, amorphous indium gallium zinc oxide (a-IGZO) has been commercialized due to its low off-state current and high field-effect mobility [1,2,3,4,5]. However, a-IGZO poses several challenges, including the high cost and limited availability of indium, as well as thermal instability owing to crystallization above 600 °C [6,7,8,9]. These drawbacks hinder its widespread application, particularly in large-area or low-temperature processes.

To overcome these limitations, nitrogen-doped zinc oxynitride (ZnON) has been proposed as a promising alternative. ZnON offers low-temperature process compatibility, reduced environmental impact, and improved electrical performances. Nitrogen doping effectively passivates oxygen vacancies (V_O_), leading to enhanced carrier mobility and stability under positive bias stress (PBS) [10,11,12,13,14]. Furthermore, ZnON exhibits a low effective mass and reduced percolation barrier effects, enabling high mobility even in amorphous or nanocrystalline forms [15,16,17,18,19]. Various post-deposition treatments have been employed to further enhance the electrical performance of oxide semiconductors, including thermal annealing, plasma exposure, and surface passivation [20,21,22]. These methods, however, often require long processing times and vacuum environments, and they lack spatial selectivity [23].

In this context, deep ultraviolet femtosecond (DUV fs) laser annealing offers a rapid, localized, and vacuum-free alternative. Owing to their ultrashort pulse duration and strong photon absorption at short wavelengths, femtosecond lasers enable precise energy delivery with minimal thermal damage [24,25,26]. In particular, DUV fs laser annealing allows for selective property modulation by digitally tuning parameters such as pulse duration, repetition rate, and scan speed [27,28,29].

In this study, we apply DUV fs laser annealing to ZnON TFTs and demonstrate selective threshold voltage modulation. The electrical, structural, and chemical changes induced by the laser process are systematically characterized. Furthermore, we demonstrate the feasibility of this approach through the fabrication and evaluation of an NMOS inverter composed of ZnON L.A. TFTs and pristine ZnON TFTs.

## 2. Experimental Methods

### 2.1. Fabrication of ZnON TFTs

The fabrication process of TFTs with a ZnON channel and indium tin oxide (ITO) source/drain (S/D) electrodes in a bottom-gate, staggered configuration is illustrated in Figure S1. Thermally oxidized SiO_2_ (thickness: 300 nm) on p^+^-Si substrates was sequentially cleaned using acetone, isopropyl alcohol (IPA), and deionized water for 10 min each using a sonication bath. The substrates were then dried on a hot plate at 100 °C to remove moisture.

The ZnON channel layer was deposited by reactive RF magnetron sputtering using a 99.99% Zn target. Deposition was carried out with a shadow mask at an RF power of 120 W, under a gas flow ratio of Ar:O_2_:N_2_ = 3:1:100 sccm, at a chamber pressure of 5 mTorr. The deposition time was 1 min, resulting in a 26 nm thick ZnON film. After the deposition, the films were annealed at 250 °C for 1 h in ambient atmosphere. Subsequently, ITO source and drain electrodes were deposited and patterned using a shadow mask. The fabricated TFTs had channel dimensions of width (W) and length (L) of 1000 μm and 100 μm, respectively.

### 2.2. Laser-Annealing Methods

DUV femtosecond laser annealing (FHG, Satsuma Display, Bordeaux, France) was selectively applied to the channel region, ensuring no overlap with the electrodes. The laser beam had a diameter of 42 μm. The laser source operated at a wavelength of 257 nm with a power of 2.2 W and a scan speed of 100 mm/s, corresponding to an energy intensity of 0.4 J/cm^2^. The pulse repetition rate was set to 100 kHz. The pulse duration was less than 350 fs, enabling ultrafast, localized processing.

### 2.3. Characteristic Measurements

Electrical characterizations, including transfer and output curves as well as gate-bias stress stability, were conducted at room temperature using a probe station and a parameter analyzer (HP-4156C). During measurements, the drain voltage (V_D_) was fixed at 10 V, while the drain current (I_D_) was measured as the gate voltage (V_G_) was swept from −20 V to 30 V.

The structural properties of the ZnON and laser-annealed ZnON (ZnON L.A.) thin films were analyzed using grazing incidence X-ray diffraction (GIXRD) using Cu Kα radiation (Rigaku, Tokyo, Japan) at an incident angle of 0.5°. Surface morphology before and after laser annealing was examined by scanning electron microscopy (SEM, Hitachi SU8600). The chemical composition and bonding states were analyzed using X-ray photoelectron spectroscopy (XPS, AXIS SUPRA, Stretford, UK) in depth profile mode on Si substrates. The optical transmittance and absorbance of ZnON L.A. films were measured using a UV–vis spectrophotometer. Ultraviolet photoelectron spectroscopy (UPS, NEXSA, Waltham, MA, USA) was used to determine the work function and the energy difference between the Fermi level and the valence band maximum (VBM). All measurements were performed before and after laser annealing to assess the effect of the DUV femtosecond laser process on the material and device properties.

## 3. Results and Discussion

**Figure 1:**
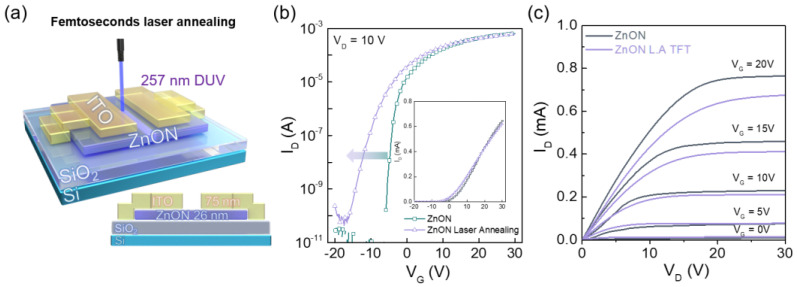
(**a**) Schematic illustration of ZnON TFT structure under DUV fs laser annealing. (**b**) Transfer characteristics of ZnON TFTs before and after laser annealing (inset: linear-scale plot). (**c**) Output characteristics of pristine and ZnON L.A. TFTs.

Figure 1a illustrates a 3D schematic of the ZnON TFT during DUV fs laser annealing, which was employed to induce a Vth shift. Figure 1b presents the transfer characteristics of the ZnON TFT before and after the laser treatment, measured with V_G_ from −20 V to 30 V under a fixed V_D_ of 10 V. Figure S2 presents the transfer characteristics of ZnON TFTs under different laser energy intensities. The data show minimal change at 0.2 J/cm^2^, a significant negative shift in off-current at 0.3 J/cm^2^, a degradation in on-current at 0.5 J/cm^2^, and a complete loss of device characteristics at 0.6 J/cm^2^. Based on these observations, 0.4 J/cm^2^ was chosen as the optimized condition. Transfer characteristics of ZnON TFTs were measured at V_D_ = 1, 2, 5, and 10 V before and after DUV femtosecond laser annealing. Transfer curves were compared before and after laser treatment to examine its effects under various drain voltage conditions (Figure S3). A slight decrease in on-current is observed in the output characteristics in Figure 1c. Table 1 shows the parameters that compare the electrical characteristics of each device. Electrical parameters, including saturation mobility (μ_sat_), Vth, on/off current ratio, and subthreshold swing (S.S), were extracted to compare the device performances. μ_sat_ and Vth were obtained from the linear-scale transfer characteristics, whereas the on/off current ratio and S.S were extracted from the logarithmic-scale data. The extracted μ_sat_ values were 22.30 and 21.09 cm^2^/V·s for the pristine ZnON TFT and ZnON L.A. TFTs, respectively, showing no significant degradation. The increased carrier concentration resulted in negative shifts in V_th_ and turn-on voltage (V_on_) by approximately 2 V and 10 V. The on/off current ratio decreased by nearly one order of magnitude, from 2.03 × 10^8^ to 1.73 × 10^7^. The S.S increased from 0.65 V/dec to 1.65 V/dec, indicating degraded switching characteristics due to an increase in the interface trap density (D_it_), which increased from approximately 7.08 × 10^11^ to 1.86 × 10^12^ cm^−2^eV^−1^, as extracted from the subthreshold swing.

**Figure 2:**
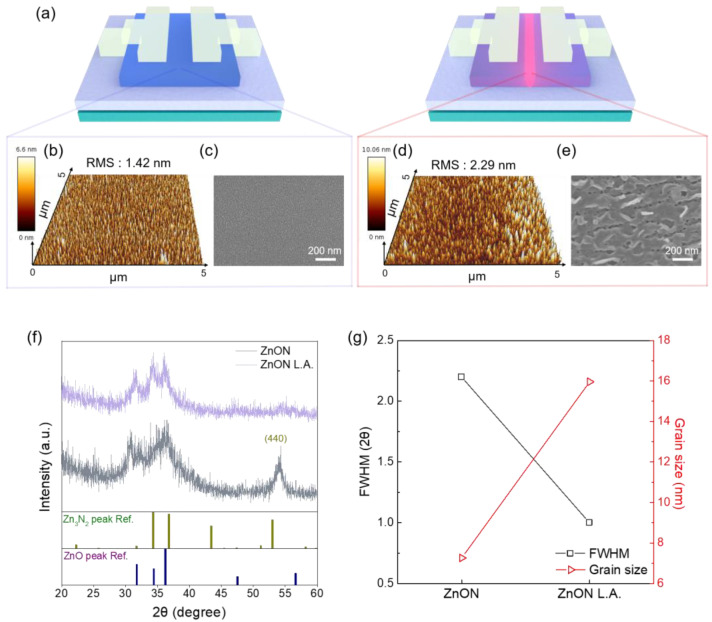
(**a**) Schematic illustration of ZnON TFT and ZnON L.A. TFT. (**b**) Surface roughness of the ZnON film analyzed by AFM, with an RMS value of 1.42 nm. (**c**) Surface SEM image of the ZnON film. (**d**) Surface roughness of the ZnON L.A. film analyzed by AFM, showing an RMS value of 2.29 nm. (**e**) Surface SEM image of the ZnON L.A. film. (**f**) X-ray diffraction (XRD) patterns of ZnON and ZnON L.A., with reference peaks of Zn_3_N_2_ and ZnO. (**g**) Grain size estimated from the FWHM of the ZnO (100) peak.

Surface morphology was analyzed using AFM and SEM to investigate potential damage induced by laser exposure. Figure 2a provides a schematic representation of the device structure before and after laser annealing. Surface roughness was quantitatively evaluated via AFM, while SEM was examined to observe morphological changes. The root mean square (RMS) roughness of the pristine ZnON thin film surface was measured to be 1.42 nm (Figure 2b), whereas the laser-annealed ZnON thin film showed an increased RMS roughness of 2.29 nm (Figure 2d). Additional planar AFM images are provided in Figure S4. SEM images clearly showed that the pristine film exhibited crystalline-like surface features (Figure 2c), while the laser-annealed film displayed signs of significant surface damage (Figure 2e) [30].

As a result, GIXRD analysis was performed to examine whether changes in surface morphology affected the physical structure of the thin film. The diffraction patterns are shown in Figure 2f, with reference peaks for Zn_3_N_2_ (PDF No. 01-088-0618) and ZnO (PDF No. 00-036-1451) included for comparison. Although distinct diffraction peaks were not prominent, likely due to low crystallinity, the presence of broad features suggests a mixture of amorphous ZnON, ZnO, and Zn_3_N_2_ phases. This is consistent with the film having been deposited via reactive sputtering under an O_2_/N_2_ gas mixture using a Zn metal target [31,32]. After laser annealing, the Zn_3_N_2_ peak at 2θ = 52.92°, corresponding to the (440) plane, disappeared, suggesting a reduction in Zn_3_N_2_ phase content and a relative enhancement of ZnO crystallinity. Given the weak intensity of the Zn_3_N_2_ at the 2θ = 31.66° of (222) reference diffraction peak, the crystallite size was calculated using the ZnO at the 2θ = 31.77° of (100) diffraction peak, based on the full width at half maximum (FWHM) and the following Debye–Scherrer Equation (1):

(1) $$D\;=\;\frac{K\lambda }{\beta \cos\theta }$$

where D is the dimension of the grain, θ is the Bragg angle, β is the FWHM, and λ is the X-ray wavelength [33]. As shown in Figure 2g, the grain size increased from 7.26 nm to 15.96 nm after laser annealing. These results from AFM, SEM, and XRD collectively confirm that the DUV fs laser treatment induces structural transformation, including surface roughness and grain growth.

**Figure 3:**
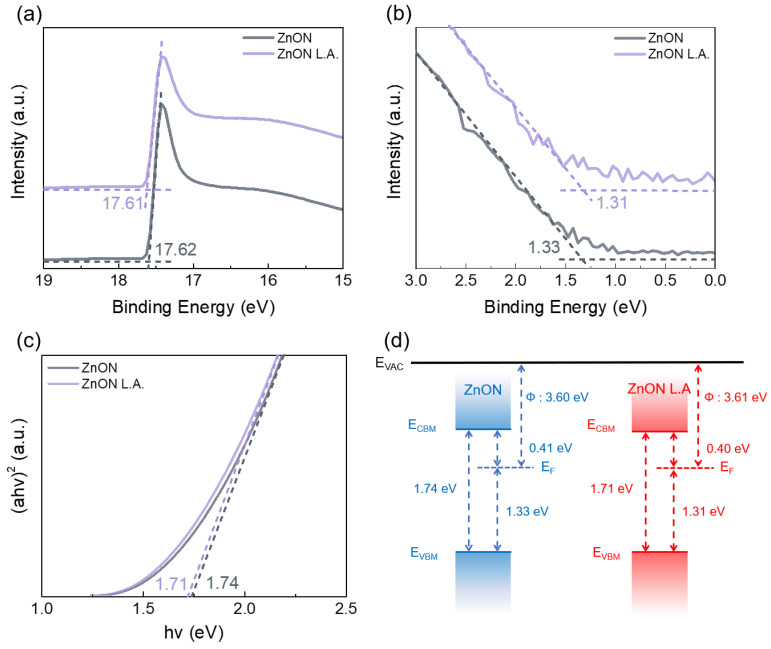
Ultraviolet photoelectron spectroscopy (UPS) and optical characterization of ZnON thin films before and after DUV fs laser annealing. (**a**) Secondary electron cutoff spectra, showing a slight shift from 17.62 eV to 17.61 eV. (**b**) Valence band edge spectra with extracted values of 1.33 eV and 1.31 eV. (**c**) Tauc plots used to determine the optical band gap, indicating a reduction from 1.74 eV to 1.71 eV. (**d**) Energy band diagram constructed from the above measurements, illustrating the electronic structure modifications induced by laser annealing.

To analyze the band structure of ZnON before and after laser annealing, ultraviolet photoelectron spectroscopy (UPS) and Tauc plot analyses were conducted. Figure 3a presents the secondary electron cutoff region obtained from the UPS spectra. By subtracting the cutoff energy from the photon energy (21.22 eV), the work function (Φ) was calculated [34], yielding values of 3.60 eV for pristine ZnON and 3.61 eV for laser-annealed ZnON. Figure 3b shows the valence band edge with respect to the Fermi level, extracted from the linear extrapolation of the UPS spectra, which indicates the energy separation between the Fermi level and the valence band maximum (VBM). Optical transmittance was measured over a wavelength range of 300–1000 nm, as shown in Figure S5a, and the corresponding absorbance spectrum is shown in Figure S5b. Using the absorbance data, the optical band gap was estimated through Tauc plot analysis (Figure 3c) [35], where linear extrapolation was used to determine the cutoff point. The extracted band gap slightly decreased from 1.74 eV (pristine) to 1.71 eV (laser-annealed). Based on the measured values of work function, VBM position, and optical band gap, energy band diagrams were constructed (Figure 3d). The overall band alignment of pristine ZnON and ZnON L.A. remained nearly unchanged. The negligible difference in energy between the Fermi level and the conduction band minimum (CBM) suggests that electron injection and transport characteristics are largely preserved after laser annealing.

**Figure 4:**
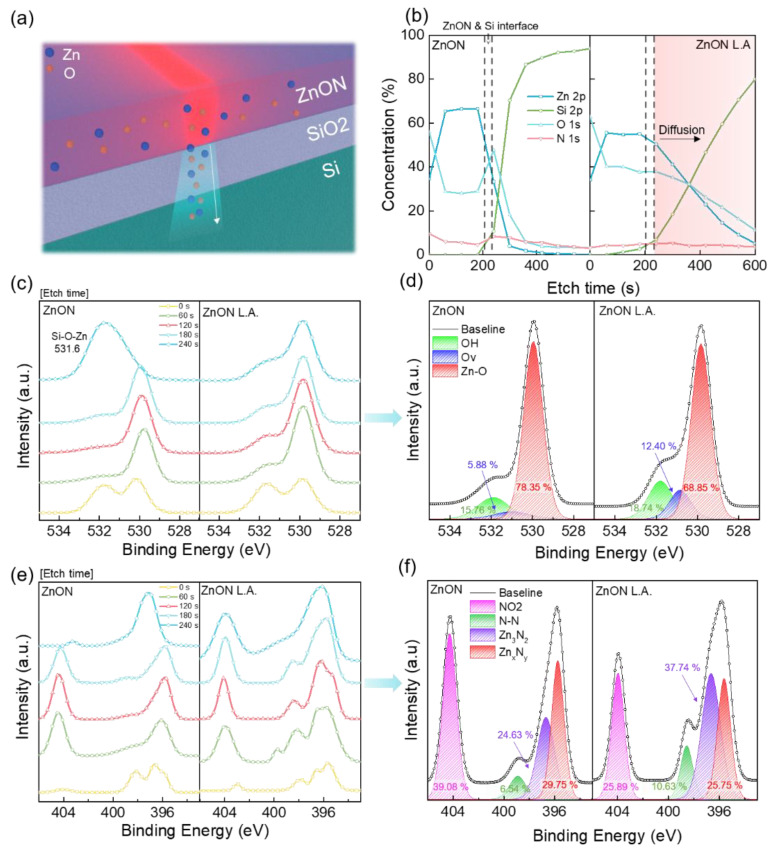
(**a**) Schematic of Zn and O atomics diffusing into the substrate. ZnON film and ZnON L.A film XPS analysis, (**b**) atomic concentration of depth profile, (**c**) XPS spectra of O 1s at different etching times (checking for tendency match), and (**d**) O 1s peak of etch time of 180 s. (**e**) XPS spectra of N 1s at different etching time and (**f**) N 1s peak at etch time of 180 s.

To investigate the changes in the chemical bonding states of ZnON before and after laser annealing, X-ray photoelectron spectroscopy (XPS) analysis was conducted. All spectra were calibrated using the standard C 1s peak at 284.6 eV [36]. Figure 4a presents a schematic illustration of Zn and O diffusion toward the substrate, which is corroborated by the depth profile shown in Figure 4b. After laser annealing, the interface between the ZnON layer and the Si substrate becomes less distinct, indicating elemental interdiffusion of Zn and O atoms. Note that although slight interfacial diffusion of Zn and O is observed after laser annealing, this diffusion is spatially confined near the interface and does not extend deeply into the dielectric layer. Given the 300 nm thickness of the gate oxide, such localized diffusion is unlikely to influence the overall dielectric properties or compromise the accuracy of the extracted electrical parameters. Figure 4c shows the evolution of the O 1s core level spectra with increased etch time. In the pristine ZnON film, a notable peak shift begins at 240 s, while the ZnON L.A. film maintains a consistent spectral profile beyond 240 s, further supporting the diffusion of elements into the substrate. The peak observed at 240 s in the pristine ZnON film corresponds to the Si–O–Zn bonds [37,38].

The relative elemental concentration presented in Figure 4b was quantified based on the peak area obtained from deconvoluted high-resolution XPS spectra using the CasaXPS software(Version 2.3.26PR1.0). Specifically, the O 1s core level spectrum was deconvoluted into three components corresponding to Zn–O bonds (lattice oxygen, 529.8 eV), oxygen vacancies (V_O_, 530.8 eV), and hydroxyl groups (OH, 531.8 eV), and the relative percentages were calculated by normalizing the area of each component to the total O 1s peak area. Additionally, the N 1s core level was deconvoluted into four peaks corresponding to NO_2_ (404.0 eV), N–N bonds (398.7 eV), Zn_3_N_2_ (396.6 eV), and Zn_x_N_y_ (395.6 eV), and their relative concentrations were calculated using the same normalization procedure. Since electron transport primarily occurs near the interface between the channel and the substrate, a detailed O 1s analysis was performed at the 180 s etch depth, which corresponds to the interfacial region. Among these, V_O_ is particularly relevant since its increase may enhance carrier concentration and mobility while also degrading device stability and reliability. The relative V_O_ content increased from 5.88% in the pristine ZnON film to 12.40% in ZnON L.A. film, suggesting a trade-off between increased carrier density and reduced reliability.

Unlike typical oxide semiconductors, ZnON also requires an analysis of nitrogen-related bonding. Figure 4e shows the depth-dependent evolution of the N 1s spectra. Similarly to the O 1s results, the ZnON L.A. film shows consistent spectral features at both 180 s and 240 s, whereas the pristine ZnON film shows spectral changes at 240 s, indicating the film–substrate boundary. Figure 4f shows the deconvolution of the N 1s spectrum at the 180 s depth, where four distinct peaks were identified. Among these, Zn_x_N_y_ species are associated with nitrogen-related defects (V_N_), while Zn_3_N_2_ bonds are related to carrier transport. After laser annealing, the proportion of Zn_x_N_y_ decreased from 29.75% to 25.75%, suggesting a reduction in nitrogen vacancies. In contrast, the Zn_3_N_2_ peak increased from 24.63% to 37.74%, indicating enhanced bonding states that could promote mobility. Despite the increase in Zn_3_N_2_ bonding, the measured field-effect mobility of ZnON L.A. decreased. This discrepancy is attributed to an increased density of interface traps. As observed in the depth profile, laser-induced changes are not confined to the channel region but also extend into the substrate, potentially affecting the gate insulator. This results in higher interface trap densities, thereby requiring stronger gate voltages to achieve carrier saturation, which explains the relatively unchanged on-current. Consequently, the increased interface trap density is expected to negatively impact long-term device stability.

**Figure 5:**
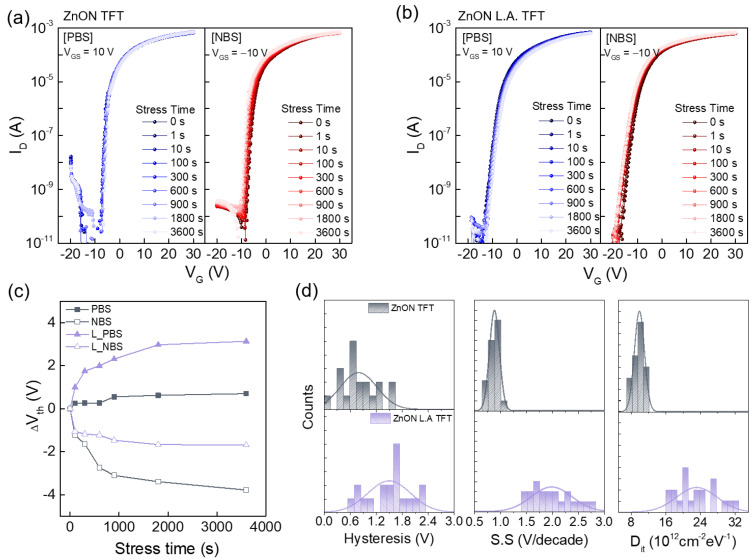
Bias stress stability analysis of (**a**) ZnON TFT and (**b**) ZnON L.A. TFT. (**c**) V_th_ shifts as a function of stress time under PBS and NBS. (**d**) Histogram statistics of hysteresis, S.S, and D_it_ values. Twenty devices were measured for statistical analysis.

Device stability under electrical stress was evaluated through bias stress testing. Figure 5a shows the results of the positive and negative bias stress (PBS and NBS) tests for pristine ZnON TFTs. The gate voltage of +10 V was applied for PBS and −10 V for NBS, each maintained for 3600 s with a constant V_D_ of 10 V. Figure 5b presents the corresponding results for ZnON L.A. TFTs under identical conditions. The overall Vth shift trends are summarized in Figure 5c. A degradation in S.S was observed in ZnON L.A. TFTs, indicating increased charge trapping near the gate dielectric/semiconductor interface, which negatively affects stability under PBS conditions [39]. In contrast, the devices demonstrated relatively stable performance under NBS conditions. The reduced ΔV_th_ under NBS for ZnON L.A. TFT is attributed to the decreased concentration of V_N_, which is ionized via hole accumulation and subsequent electron release at the interface [40,41]. These results suggest that laser annealing suppresses free electron generation under NBS, while enhancing negative charge trapping under PBS conditions [42,43]. Figure 5d presents histograms of hysteresis (∆V_th_), S.S, and D_it_ values extracted from 20 devices. As expected, hysteresis increased in ZnON L.A. due to the elevated D_it_, leading to a larger ∆V_th_. The S.S values, calculated as the inverse slope of the transfer curve, also confirmed this degradation. The interface trap density was calculated using Equation (2):

(2) $$D_{\mathrm{it}}\;=\left[\frac{\mathrm{SS}q\log\left(e\right)}{kT}-1\right]\frac{C_{i}}{q}$$

where *q* is the elementary charge, *k* is Boltzmann’s constant, and *C_i_* is the oxide capacitance [44]. The calculated results confirm that D_it_ increases after laser annealing, which contributed to reduced stability under PBS conditions.

**Figure 6:**
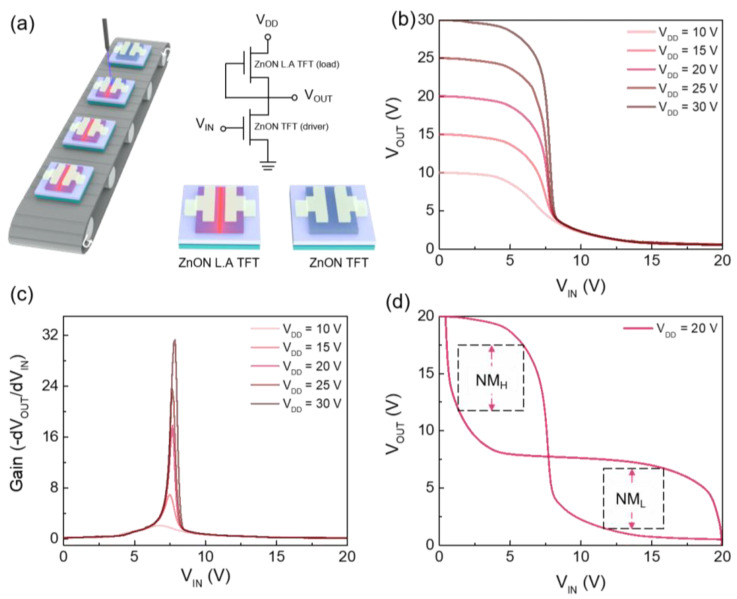
(**a**) Schematic illustration of the laser-annealing process and the NMOS inverter circuit configuration. (**b**) VTC for the NMOS inverter with the V_DD_ of 10–30 V, (**c**) corresponding voltage gain characteristics, and (**d**) butterfly curve used for noise margin evaluation at V_DD_ = 20 V.

Finally, an NMOS inverter was fabricated using ZnON TFT and ZnON L.A. TFT. As illustrated in Figure 6a, the ZnON L.A. TFT serves as the load, while the pristine ZnON TFT is used as the driver. The devices were fabricated on separate substrates and electrically connected via external wiring. The corresponding circuit diagram is depicted in Figure 6a. Figure 6b presents the voltage transfer characteristics (VTC) of the inverter, measured by sweeping the input voltage (V_IN_) from 0 V to 20 V under supply voltages (V_DD_) ranging from 10 V to 30 V. Based on the VTC curve, the voltage gain and noise margin were evaluated. The voltage gain, defined as the maximum slope of the VTC curve, is plotted in Figure 6c, with a peak gain of 31 observed at V_DD_ = 30 V. Noise margins were extracted from the VTC at V_DD_ = 20 V, as shown in Figure 6d [45]. The high and low noise margins (N_MH_ and N_ML_) were calculated as N_MH_ = V_OH_ − V_IH_ = 5.84 V and N_ML_ = V_IL_ − V_OL_ = 5.34 V, corresponding to 58% and 53% of V_DD_/2, respectively. These values are indicative of stable inverter operation with clear logic level distinction. The results highlight the feasibility of applying the demonstrated logic implementation to future integrated systems, including selectively annealed CMOS inverters, gas sensors, memory arrays, and OLED-based mesh electronics.

## 4. Conclusions

In this study, DUV fs laser annealing was applied to the channel region of ZnON TFTs, resulting in a negative shift in V_th_. A key advantage of this approach is its ability to selectively modify electrical characteristics without requiring vacuum environments. Using a single material and a one-step process, V_th_ modulation was successfully achieved within 100 microseconds. To investigate the physical and chemical changes induced by laser annealing, XRD, SEM, AFM, UPS, and XPS analyses were conducted before and after treatment. Depth profiling of the XPS spectra confirmed that Zn and O atoms diffused toward the substrate. A distinct variation in elemental distribution was observed between etching times of 180 s and 240 s, indicating depth-dependent changes resulting from the annealing. The stability of the TFTs showed no significant degradation after annealing, demonstrating that the devices remain suitable for practical applications. Furthermore, the inverter exhibited a noise margin exceeding 50%, verifying its functional operation. Overall, the feasibility of selective annealing within short processing times was demonstrated, suggesting broader applicability to logic circuits, fine patterning, and various advanced electronic applications.

## Figures and Tables

**Table 1:** Comparison of key electrical properties of the ZnON TFT and ZnON L.A. TFT.

Parameter	μ_sat_ [cm^2^/Vs]	Vth [V]	On/Off Ratio	S.S [V/Dec]	Dit [cm^−2^eV^−1^]
ZnON TFT	22.30	−3.75	2.03 × 10^8^	0.65	7.08 × 10^11^
ZnON L.A. TFT	21.09	−5.80	1.71 × 10^7^	1.61	1.86 × 10^12^

## Data Availability

Data is contained within the article.

## Supplementary Materials

The following supporting information can be downloaded at https://www.mdpi.com/article/10.3390/nano15161247/s1, Figure S1: TFT fabrication process; Figure S2: Laser annealing optimization; Figure S3: TFT transfer curve properties according to V_D_; Figure S4: AFM planar image; Figure S5: transmittance and absorbance of ZnON and ZnON L.A. film.
