# Supplementary material for: Selective DUV Femtosecond Laser Annealing for Electrical Property Modulation in NMOS Inverter
Source: Nanomaterials (Basel). 2025 Aug 14;15(16):1247. doi: 10.3390/nano15161247 (PMC12388412; doi:10.3390/nano15161247)
Supplement: Supplementary file 1 [file nanomaterials-15-01247-s001.zip › nanomaterials-3795486-supplementary.pdf]

Selective DUV Femtosecond Laser Annealing for Electrical Property Modulation in NMOS Inverter

Joo Hyun Jeong[1], Won Woo Lee[1], Sang Jik Kwon[2], Min-Kyu Park[1*], and Eou-Sik Cho[1,2,*]

[1]Dept. of Semiconductor Engineering, Gachon University, Seongnam City, Gyeong-gi 13120, Korea
[2]Dept. of Electronic Engineering, Gachon University, Seongnam City, Gyeong-gi 13120, Korea

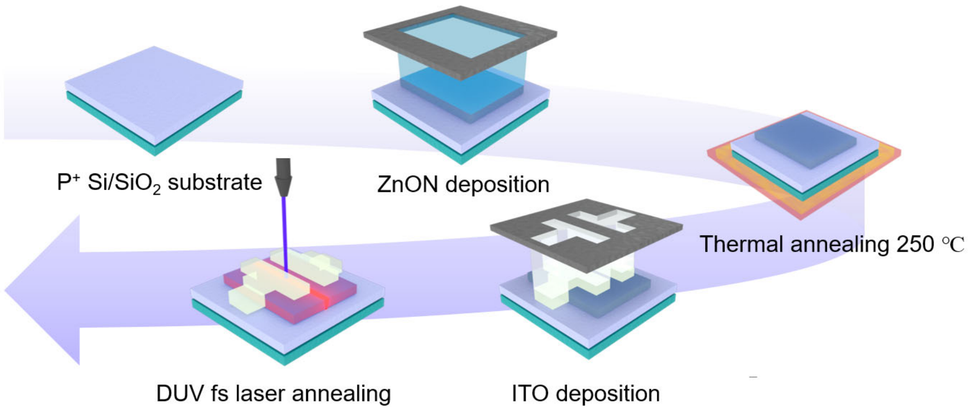

Figure S1. ZnON based TFT Fabrication and DUV fs laser annealing process schematic.

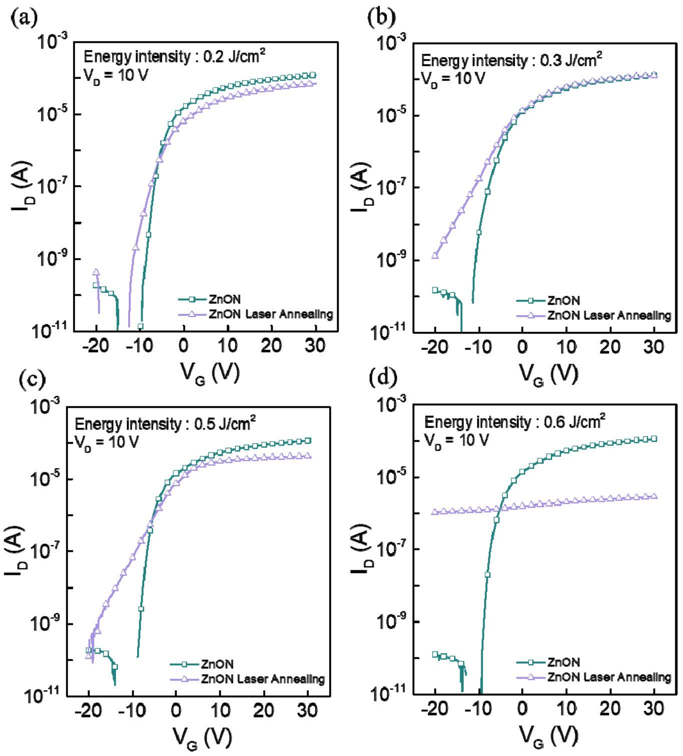

Figure S2. Optimization of laser annealing energy intensity for ZnON TFTs. Transfer characteristics measured under varying laser energy intensities: (a) 0.2 J/cm² showing negligible change; (b) 0.3 J/cm² exhibiting a significant negative shift in the off-current; (c) 0.5 J/cm² showing notable degradation in on-current; and (d) 0.6 J/cm² where the transistor characteristics are completely lost.

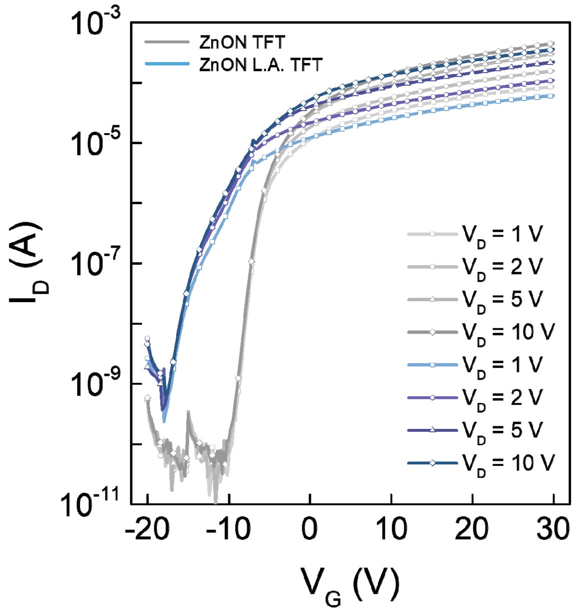

Figure S3. Transfer characteristics of ZnON TFTs measured at V_D=1, 2, 5, and 10 V before and after DUV femtosecond laser annealing. The measurements were performed to compare the effects of laser treatment under various drain bias conditions.

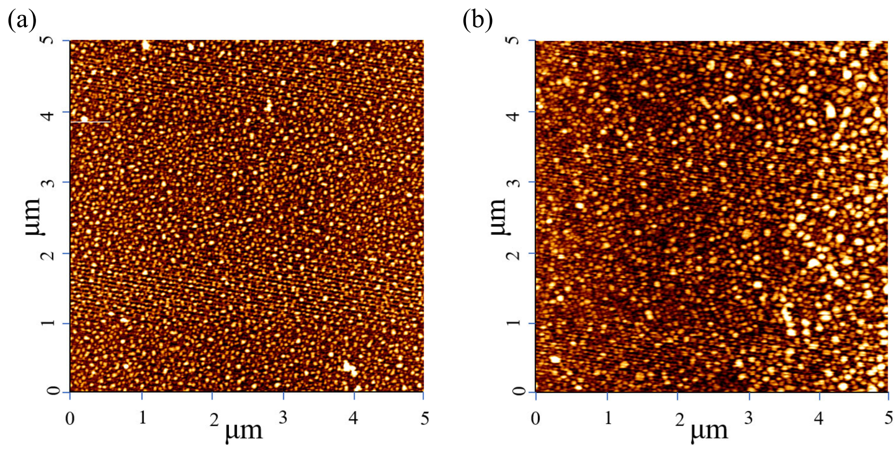

Figure S4. Planar image showing surface roughness of AFM analysis (a) before and (b) after DUV fs laser annealing

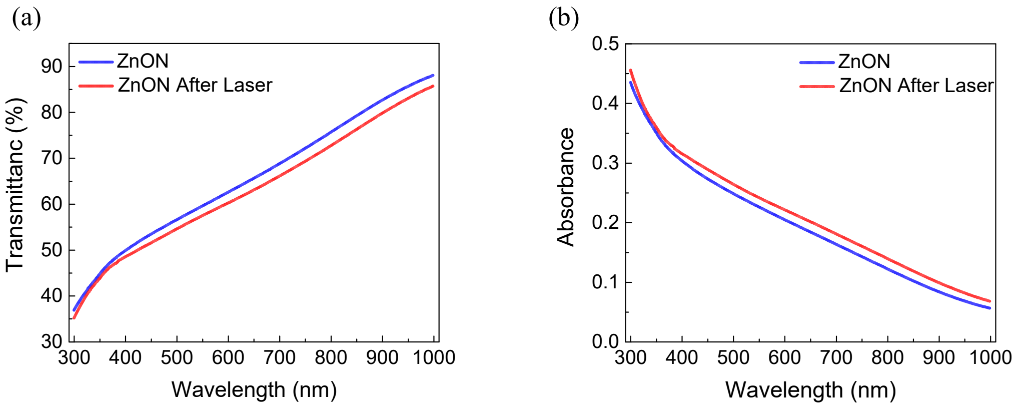

Figure S5. (a) Transmittance and (b) absorbance of ZnON thin films annealed using a 257 nm DUV fs laser annealing.
